# Preparation of Coated Valproic Acid and Sodium Valproate Sustained-release Matrix Tablets

**DOI:** 10.4103/0250-474X.65026

**Published:** 2010

**Authors:** T. Phaechamud, W. Mueannoom, S. Tuntarawongsa, S. Chitrattha

**Affiliations:** Department of Pharmaceutical Technology, Faculty of Pharmacy, Silpakorn University, Nakhon, Pathom-73000, Thailand

**Keywords:** Coated matrix, preparation technique, sodium valproate, sustained-release, valproic acid

## Abstract

The aim of this research was to investigate the technique for preparation of coated valproic acid and sodium valproate sustained-release matrix tablets. Different diluents were tested and selected as the effective absorbent for oily valproic acid. Effect of the amount of absorbent and hydroxypropylmethylcellulose on drug release from valproic acid-sodium valproate matrix tablets prepared with wet granulation technique was evaluated in pH change system. Colloidal silicon dioxide effectively adsorbed liquid valproic acid during wet granulation and granule preparation. The amounts of colloidal silicon dioxide and hydroxypropylmethylcellulose employed in tablet formulations affected drug release from the tablets. The drug release was prominently sustained for over 12 h using hydroxypropylmethylcellulose-based hydrophilic matrix system. The mechanism of drug release through the matrix polymer was a diffusion control. The drug release profile of the developed matrix tablet was similar to Depakine Chrono^®^, providing the values of similarity factor (*f*2) and difference factor (*f*1) of 85.56 and 2.37, respectively. Eudragit^®^ L 30 D-55 was used as effective subcoating material for core matrix tablets before over coating with hydroxypropylmethylcellulose film with organic base solvent. Drug release profile of coated matrix tablet was almost similar to that of Depakine Chrono^®^.

Matrix diffusion is a suitable system in producing oral sustained release dosage form, especially tablets. Matrix tablet can be achieved by using appropriate type and concentration of a matrix substance, followed by general manufacturing process mainly including granulation and compression. Hydroxypropylmethylcellulose (HPMC) is the major hydrophilic carrier material used for the preparation of oral controlled drug delivery systems. One of its most important characteristics is the high gelation velocity and viscosity, which has a significant effect on the release kinetics of the incorporated drug[1,2]. It was proven that HPMC at high concentration promoted the drug release approaching to a zero order release kinetic because of its gelation properties[2]. Colloidal silicon dioxide such as Aerosil^®^ 200 has been used in several pharmaceutical applications such as a moisture adsorbent, free-flow agent and glidant in the tablet manufacturing[3]. In theophylline-loaded lipid microparticles, Aerosil^®^ 200 was employed as a thickening and a suspending agent[4].

Valproic acid (VA) and sodium valproate (VAS) are anticonvalsants widely used for treatment of simple and complex absence seizures. Physical characteristic of VA are as follows; clear, colorless to pale yellow, slightly viscous liquid, and sparingly soluble in water. The solubility data are 1.27 mg/ml in water and 1.25 mg/ml in 0.1N HCl. Boiling point of VA is 221-222°. VA is a very stable compound since no degradation is observed by the action of heat, light, and strong aqueous alkali, or acid[5]. VAS is a white crystalline, very hygroscopic powder and very soluble in water and alcohol[6]. One gram of VAS is soluble in 0.4 ml of water and also in 1.5 ml of ethanol. VAS was extremely stable when it was refluxed in water, 1.0 N hydrochloric acid, or 1.0 N sodium hydroxide for 3 h. Also, it was very stable when subjected to heat at 110° for 10 days and to sunlight for 30 days in the dry environment. The pKa values of VA and VAS are 4.6 and 4.8, respectively[5].

VA and VAS have been used in combination because there are minor differences in the pharmacokinetics of the formulation and accessibility in market[7]. VA and VAS are available in different dosage forms; capsule, tablet, enteric-coated tablet, sprinkle, liquid, intravenous, suppository and controlled-release formulations[8]. Sustained-release formulation of the combination between VA and VAS reduces the fluctuation in plasma drug concentrations, thus minimizing or preventing plasma peak-related adverse events, and allows prolongation of the dosing interval enabling a once or twice daily administration with inherent benefits in terms of patient compliance[9]. Due to oily characteristic of VA, tablet formulation is difficult to prepare. Divalproex sodium, a compound containing an equal proportion (on a molar basis) of sodium valproate and valproic acid, dissociates into valproate ion in the gastrointestinal tract[10]. Divalproex sodium requires twice or three times daily administration. Once-daily administration of divalproex sodium extended-release tablets may potentially be used to sustain plasma valproic acid concentrations within the usually accepted therapeutic ranges for various indications in children and aldolescent[11]. Controlled-release of divalproate sodium tablet could provide desired nearly constant therapeutic plasma concentration over the entire 24 h dosing interval[10]. A relatively good correlation was observed between the absorption profiles and the dissolution profiles of the developed 200 mg and 400 mg VAS sustained-release tablet by a membrane-controlled system[12]. An addition of citric acid in the film coat exerted a plasticizing effect on the enteric polymer film and improved film formation and polymer coalescence. As citric acid was greater than 10% (w/w) in the enteric coated VAS pellets, a decrease in drug content was observed due to the conversion of sodium valproate to the volatile compound, valproic acid[13]. However, technique concentrating on matrix preparation and coating of VA-VAS tablet has not been reported.

The purpose of this research was to study the technique for the preparation of coated VA and VAS sustained-release matrix tablets, using HPMC as matrix former by wet granulation technique and to compare drug release of the developed tablets to that of a commercial product, Depakine Chrono^®^. The effect of excipients on physical properties and drug release from matrix tablet was also investigated.

## MATERIALS AND METHODS

Valproic acid (Lot 041101) and sodium valproate (Lot 040901E) were purchased from Hunan Xiangzhong Pharmaceutical Co., Ltd., Shaoyang Hunan, China. Valproic acid reference standard (Lot 123K3748, Sigma, Taufkirchen, Germany) was used as received. Colloidal silicon dioxide (Aerosil^®^ 200, Degussa, Dusseldorf, Germany), hydroxypropylmethylcellulose (Methocel^®^ K 15 M, Dow Chemical, The heeren, Sigapore) and microcrystalline cellulose (Avicel^®^ PH 102, FMC Biopolymer, Philadelphia, USA) were used as matrix components. Isopropyl alcohol (Shell Chemicals, Sereya, Singapore) was used as the granulating liquid. Povidone (Plasdone^®^ K 90, ISP technologies, Texas, USA) and magnesium stearate (Nof corporation, Tokyo, Japan) were used as binder and lubricant, respectively. The coating material was hydroxypropylmethylcellulose (Pharmacoat^®^ 615, Shin-Etsu chemical Co., Ltd, Tokyo, Japan) and Eudragit^®^ 30D-55 which was purchased from Rama Production, Bangkok, Thailand. Triethyl citrate (Lot AG CH9470, Fluka Chemical, Buch, Switzerland) was used as plasticizer. Titanium dioxide (Sensient, Scarlino, Italy) and talcum (Super^®^-1250) (Shengtai Chem Co., Ltd., Guangdong, China) were also added as opacifier in coating material. Isopropyl alcohol (Shell Chemicals, Sereya, Singapore) and methylene chloride (DOW Chemical, The heeren, Singapore) were used as solvents in the coating process. Calcium carbonate (Fujian Sannong Calcium Carbonate Co., Ltd., Sanming, Fujiang, China), corn starch (Weifang S Co., Ltd., Shahengtai Medicine Co., Ltd., Shandong, China) and dibasic calcium phosphate (Yichang Shenfa Foreign Trade Co., Ltd., Shanghai, China) were used as received.

### Adsorption of VA with some excipients:

VA is an oily liquid which is difficult for applying in tablet preparation. Colloidal silicon dioxide, talc, microcrystalline cellulose, calcium carbonate, corn starch and dibasic calcium phosphate were individually tested for VA adsorption by mixing with VA 145 mg. Each of these excipients was gradually weighed for mixing with VA using mortar and pestle until VA was completely adsorbed with no liquid residue left (n=3).

### Preparation and evaluation of matrix granule:

The matrix granules were prepared by wet granulation method. VA was gradually adsorbed on colloidal silicon dioxide (Aerosil^®^ 200) using the mortar and pestle. The amount of colloidal silicon dioxide used in this study was varied (7, 9, 10, 15 and 20 % by weight). Microcrystalline cellulose and hydroxypropylmethylcellulose (Methocel^®^ K15M) of 0, 5, 7.5, 10, 12.5, 15 and 20 % by weight were dried, mixed and screened through a 20-mesh sieve, then mixed with the active ingredient. Wet granules were prepared by adding PVP-K 90 in isopropyl alcohol solution (3% w/w of total weight of core tablet formulation) into powder mixture, sheared by the pestle and screened through a 12-mesh sieve. The granules were tray dried at 60° using a hot air oven for 3 h. The dried granules were screened through a 20-mesh sieve before the evaluation of flow property and compressibility. The humidity in a granule-preparation room was regulated around 50 % RH. The bulk and tapped densities of the granules were determined in triplicate using the test for apparent volume, and the Carr's index was calculated. The amount of ingredients used in each formulation (presented as SR1 to SR 12) was shown in the Table 1.

**TABLE 1:** FORMULA OF VALPROIC ACID AND SODIUM VALPROATE SUSTAINED-RELEASE MATRIX TABLETS

Ingredient	Formula (amount per tablet, mg)											
	SR1	SR2	SR3	SR4	SR5	SR6	SR7	SR8	SR9	SR10	SR11	SR12
VA	145	145	145	145	145	145	145	145	145	145	145	-
VAS	333	333	333	333	333	333	333	333	333	333	333	500
Colloidal silicon dioxide	49	63	70	105	140	63	63	63	63	63	63	63
HPMC	70	70	70	70	70	-	35	52.5	87.5	105	140	105
Povidone	21	21	21	21	21	21	21	21	21	21	21	21
Microcrystalline cellulose	68	54	47	12	-	124	89	71.5	36.5	19	-	-
Magnesium stearate	7	7	7	7	7	7	7	7	7	7	7	7
Talc	7	7	7	7	7	7	7	7	7	7	7	7
Total	700	700	700	700	700	700	700	700	700	700	716	703

### Preparation of core matrix tablet:

VA and VAS sustained release tablets were HPMC-based hydrophilic matrix system. After the dried granules were screened through a 20-mesh sieve, they were mixed with magnesium stearate and talcum. Then the core matrix tablets were compressed using single punch tablet machine with a caplet punch (Yeoheng, Bangkok, Thailand). The 200 tablets per batch size were prepared for tablet evaluation. The process for scale up the core matrix tablet was similar to the above mention, except the amount of tablet was 2,000 tablets per batch size. The humidity in a core-matrix-tablet preparation room was regulated around 50 % RH.

### Preparation of film coated matrix tablet:

The VA and VAS sustained release core tablets were coated with film coater (model 0603/1017, N. R. Industries Co., Ltd., Bangkok, Thailand) using HPMC-based film with different thickness by varying spraying duration (1, 2, 3 and 4 h). The coating solution was prepared by adding 10% HPMC, 5% talcum and 5% titanium dioxide into a mixture of 1:1 isopropyl alcohol and methylene chloride. The conditions for coating were as follows: inlet air temperature, 60°; atomizing air pressure, 300,000 Pa; pan speed, 8 rpm and coating time, 1, 2, 3 and 4 h.

### Chromatographic condition of HPLC analysis:

A Shimadazu HPLC system model SPD-M10Avp consisting of pump, LC-10Advp (Liquid chromatograph), autosampler, SIL-10Advp, column heater, CTO-10Asvp (column oven) and detector, SPD-M10Avp (diode array detector), injection valve equipped with auto-injector, variable wavelength detector set at 220 nm and 20 microclines loop injection valve (Shimadzu, Kyoto, Japan) was used for determination of drug content. For analysis, a reversed-phase Innersole ODS 3 C_18_ (5 µm) 4.6×150 mm column was eluted by using a mixture of acetronitrile and a 0.32% W/V solution of potassium dihydrogen orthophosphate (60:40) adjusted to pH 3 with orthophosphoric acid as the mobile phase with a flow rate of 1 ml per minute and a detection wavelength at 220 nm. Quantification of VA was carried out by measuring the peak areas in relation to those of standard chromatograph analyzed under the same conditions. The VAS was converted to the free acid at this pH during the HPLC analysis. The HPLC analysis was validated for accuracy, linearity and precision before used. The correlation coefficients from the system validation for accuracy and linearity were 0.9998 and 0.9998, respectively. The precision was expressed in terms of relative standard deviation (%RSD) values. RSD values for precision were less than 2.0%, indicating a good repeatability.

### Evaluation of matrix tablets:

The hardness of tablets was determined using a hardness tester (model TBH210TD, Pharmatest, Ontario, Canada). The tablet thickness was measured using a thickness tester (Teclock, Kyoto, Japan). Friability of prepared tablets was evaluated using a friability tester (Yeo Heng, Bangkok, Thailand). In this study, the tablets were prepared by controlling the weight within 700±5% mg per tablet, hardness in the range of 127-147 N and friability no more than 0.1%. The suitable formulations were chosen for scale up and film coating. Content uniformity was determined using a HPLC method. Dissolution profiles of the prepared VA and VAS sustained release tablets were compared with that of Depakine Chrono^®^. *In vitro* dissolution testing of VA and VAS sustained release tablets was determined using a USP apparatus II dissolution tester (model VK7010, Vankel, NJ, USA), operating at 100 rpm. Dissolution test was performed in 500 ml of 0.1 N HCl for 45 min followed by 900 ml of 0.05 M phosphate buffer pH 5.5 containing 0.5% SLS, the medium temperature was maintained at 37±0.5°. The 10 ml of dissolution medium were withdrawn at 30 min, 1, 2, 3, 4, 5, 6, 8, 10 and 12 h. The medium was replenished with 10 ml of fresh buffer each time. Each sample was filtered through Nylon filter 0.45 micron. The samples were assayed by HPLP under the above mentioned analysis condition. The obtained dissolution profiles were compared with that of the Depakine Chrono^®^.

### Determination of surface morphology of matrix tablet:

The surface and cross-sectional topography of the prepared matrix tablets and Depakine Chrono^®^ were determined using a scanning electron microscope (SEM) (Maxim 200 Camscan, Cambridge, England) operated at an accelerating voltage of 20 KeV. The samples were stuck on a metal stub using carbon double adhesive and sputter coated with gold before test.

### Evaluation of similarity factor and difference factor of release profiles:

The similarity and difference of release profiles of the developed formulation was compared to that of the commercial formulation in terms of similarity factor (*f*_2_) and difference factor (*f*_1_) using the following eqns; *f*_2_ = 50×log[{1+1/n Σ_t=1_ |R_t_ – T_t_|^2^}^-0.5^×100]..1 and *f*_1_ = [{Σ_t=1_ |R_t_ – T_t_|}/{Σ_t=1_R_t_}]×100..2, where R_t_ and T_t_ are the percent drug dissolved at each time point for the sample and reference products, respectively, n is the number of dissolution sample times, and t is the time sample index[14]. The two curves are considered to be similar when *f*_2_ value is close to 100 (50-100). Release profiles are considered to be different when *f*_1_ value is close to 15, generally *f*_1_ value of less than 15 (0-15) indicates similarity between the profiles.

### Dissolution profile fitting:

Least square fitting the experimental dissolution data (cumulative drug release >10% and up to 80%) to the mathematical equations (power law, first order, Higuchi's and zero order) was carried out using a nonlinear computer programme, Scientist for Windows, version 2.1 (MicroMath Scientific Software, Salt Lake City, UT, USA). The coefficient of determination (r^2^) was used to indicate the degree of curve fitting. Goodness-of-fit was also evaluated using the Model Selection Criterion (MSC)[15], given

$$\mathrm{as}:\;\mathrm{msc}\;=\;\mathrm{In}\left\{\frac{\sum_{i=1}^{n}w_{i}\;{\left(Y_{{\mathrm{obs}}_{i}}\;-\;{\bar{Y}}_{{\mathrm{obs}}_{}}\right)}^{2}}{\sum_{i=1}^{n}w_{i}{\left(Y_{{\mathrm{obs}}_{i}}\;-\;Y_{{\mathrm{cal}}_{i}}\right)}^{2}}\right\}-\;\frac{2p}{n},\;\mathrm{where}\;Y_{\mathrm{obsi}}\;\mathrm{and}\;Y_{\mathrm{cali}}$$

are observed and calculated values of the i-th point, respectively, and w_i_ is the weight that applies to the i-th point, n is number of points and p is number of parameters.

## RESULTS AND DISCUSSION

The amount of different excipients that could adsorb 145 mg VA was 30±2.58, 180±1.67, 260±2.55, 370±1.96, 600±2.33 and 760±2.68 mg for colloidal silicon dioxide, talc, microcrystalline cellulose, calcium carbonate, corn starch and dibasic calcium phosphate, respectively. Colloidal silicon dioxide demonstrated the good adsorbent for VA, because of its fine particle about 7-40 nm in size, anomalous surface area and high silanol groups on the surface particle[16]. The silanol groups of colloidal silicon dioxide should potentially form a network structure through interparticular hydrogen bonds between the carboxyl groups of VA. Such bonding between colloidal silicon dioxide and lipid has been previously mentioned[4].

Owing to high moisture absorption ability of sodium valproate[6], the humidity in the preparation room was controlled to be less than 50% RH. The Carr's index of each granule formulation was in a range of 5-15, corresponding to the excellent flowability. The angle of repose of each granule formulation was less than 25, indicating the excellent flowability, except the angle of repose of SR11 which was in the range of 25-30, corresponding to good flowability (data not shown).

**Fig. 1:**
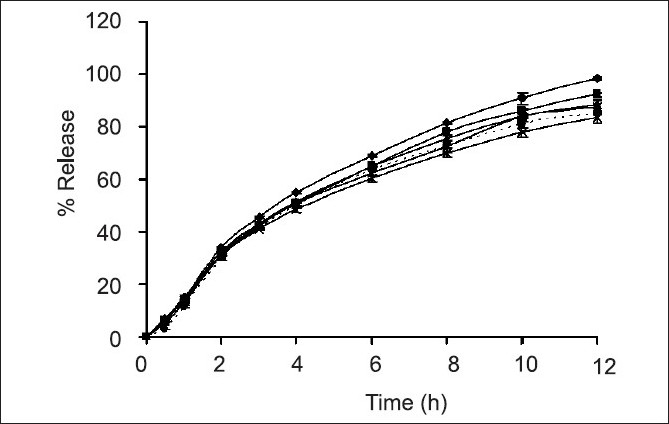
Comparative dissolution profiles. Comparative dissolution profiles of drug released from ‐‐♦‐‐ SR1; ‐‐■‐‐ SR2; ‐‐▲‐‐ SR3; ‐‐×‐‐ SR4; ‐‐×‐‐ SR4; ‐‐*‐‐ SR5; ‐‐■‐‐ Depakine ChronoR in phosphate buffer pH 6.2 (n=3)

Weight, hardness and friability of each matrix tablet were carefully controlled in a range of conditions during the tablet preparation. The label amount of drug in the prepared tablets was varied from 98.48±1.55% to 106.8±2.45%. Effect of colloidal silicon dioxide on physical properties of matrix tablets was evident. The core matrix tablet of SR2 which contained 9% colloidal silicon dioxide exhibited good appearance, non-sticking and good compressibility. On the other hand, the core matrix tablet containing less than 9% colloidal silicon dioxide exhibited sticking tablets. As the concentration of Aerosil^®^ 200 was increased from 7% to 20% by weight, the percent cumulative drug release was slightly decreased (fig. 1).

Silanol groups on the particle surface of Aerosil^®^ 200 could interact via hydrogen bond with each other to form connecting bridge. The binding ability of colloidal silicon dioxide particles promoted the drug adsorption on the surfaces[17] and the drug release was retarded. The adsorption of ketoprofen to colloidal silicon dioxide and thereafter the retardation of drug release from gel system have been reported[18]. In addition, some investigators also reported the gelation properties of colloidal silicon dioxide[19]. Due to the -OH groups on the microparticle surface, Aerosil^®^ 200 could form a great number of hydrogen bonds with dissolution medium. The gelation ability was greater when the concentration of Aerosil^®^ 200 was increased, therefore the adsorbed VA could gradually diffuse from the gel layer to dissolution medium and the drug release was slightly prolonged.

**Fig. 2:**
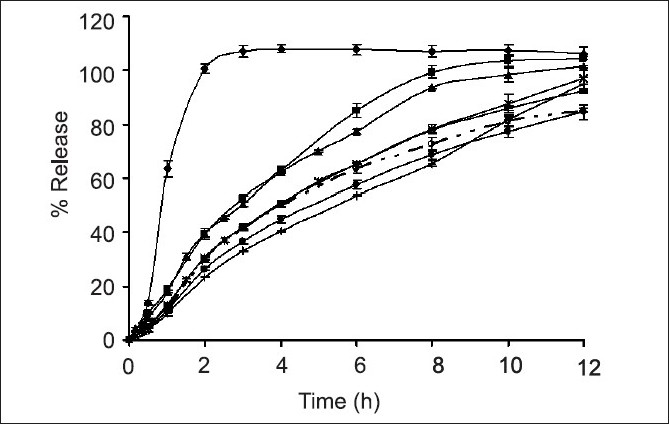
Comparative dissolution profiles. Comparative dissolution profiles of drug released from ‐‐‐‐ SR2; ‐‐♦‐‐ SR6; ‐‐●‐‐ SR7; ‐‐●‐‐ SR8; ‐‐*‐‐ SR9; ‐‐■‐‐ SR10; ‐‐+‐‐ SR11; ‐‐◊‐‐ SR12; ‐‐○‐‐ Depakine Chrono^®^ in phosphate buffer pH 6.2 (n=3)

The HPMC-based hydrophilic matrix system could prolong the drug release. As the concentration of HPMC K15 M was increased from 0 % (SR 14) to 20 % (SR 11), the drug release rate was gradually decreased (fig. 2). After the core matrix tablet initially contacted with the dissolution medium (0.1 N HCl solution), VAS, which was a water soluble drug depositing on surface matrix tablet, could be rapidly dissolved and converted to VA. Then, water penetrated the matrix, leading to polymer swelling and drug dissolution. Therefore, the drug could gradually diffuse from the matrix. With a higher polymer concentration, the resultant gel layer would be more viscous[19] and the tightness of the swollen hydrogel network was increased[20]. Therefore, VA diffusion through a gel layer to a dissolution medium was decreased. A similar result was reported on the tetracycline hydrochloride released from hydrophilic matrix systems containing HPMC K4M[21].

Dissolution profiles of all developed matrix tablets were compared to that of the commercial product, Depakine Chrono^®^, as presented in (figs. 1 and 2 and Table 2. The similarity factor (*f*_2_) values were found to be greater than 50 for most of the developed formula, except SR6, SR7, SR8 and SR12 containing 0%, 5%, 7.5% and 15% HPMC K 15 M by weight, respectively, and VAS 500 mg. Therefore, the dissolution profiles of SR6, SR7, SR8 and SR12 were different from that of Depakine Chrono^®^.

**TABLE 2:** DIFFERENCE FACTOR (*F*_1_) AND SIMILARITY FACTOR (*F*_2_) OF DISSOLUTION PROFILES FOR DEPAKINE CHRONO® AND DIFFERENT CORE MATRIX TABLETS

Formula	Difference factor (*f*_1_)	Similarity factor (*f*_2_)
SR 1	13.31	56.75
SR 2	5.82	71.20
SR 3	4.79	79.05
SR 4	4.36	79.92
SR 5	3.66	81.71
SR 6	87.51	16.44
SR 7	31.51	38.61
SR 8	25.40	43.67
SR 9	6.88	64.72
SR 10	6.81	70.31
SR 11	12.45	57.25
SR 12	39.04	34.14

Difference factor (*f*_1_) values were found to be less than 15 for most of the developed formulations, except SR6, SR7, SR8 and SR12 Table 2. SR 6 released the drug rapidly since it lacked the swellable matrix agent. SR7 and SR8 contained low concentration of swellable matrix agent, therefore the low gel layer formation and gel strength promoted a rapid erosion of the matrix[21] resulting in a rapid diffusion of drug through the dissolution medium occurred. The drug release from SR12 was faster than SR10 and Depakine Chrono^®^. Since VAS is water soluble and is not adsorbed by Aerosil^®^ 200, the conversion of VAS to VA could not promote the adsorption the employed Aerosil^®^ 200. Therefore, VAS could convert to VA rapidly and diffuse through a gel layer to the dissolution medium.

The most suitable formulae were SR3 and SR10 since the difference factor (*f*_1_) values were 4.79 and 6.81, respectively, and the values of similarity factor (*f*_2_) were 79.05 and 70.31, respectively. These systems were chosen for scale up and film coating studies. The dissolution profile of each scale-up core formulation was compared to that of pre-scale up core formulation. The physical properties of core matrix tablets after scale up were not different from the pre-scale up core tablets (data not shown).

SR3 was more suitable than SR4 and SR5 although the difference factor (*f*_1_) of SR3 was greater than that of SR4 and SR5 and the similarity factor (*f*_2_) of SR3 was less than that of SR4 and SR5. Because the amount of Aerosil^®^ 200 for SR4 and SR5 was rather high and bulky, the tablet preparation was difficult. The dissolution profile of SR3 core after scale up was similar to that of the pre-scale up core. The drug release of SR10 core after scale up was slightly faster than that of the pre-scale up core.

Both scale-up cores (SR3 and SR10) demonstrated the dissolution profiles similar to Depakine Chrono^®^ Table 3. The values of difference factor (*f*_1_) were 6.53 and 2.37, and the values of similarity factor (*f*_2_) were 68.42 and 85.56, respectively, for SR3 and SR10. Drug release from the scale up SR3 core was faster than that of the scale up SR10 core. Since the content of swellable matrix agent of the scale up SR3 core was less than that of the scale up SR10 core, therefore, the diffusion path length for the drug diffusion of the former was shorter[22].

**TABLE 3:** DIFFERENCE FACTOR (*F*_1_) AND SIMILARITY FACTOR (*F*_2_) OF DISSOLUTION PROFILES FOR DEPAKINE CHRONO® AND DIFFERENT CORE AFTER SCALE UP AND FILM COATING

Formula	Difference factor (*f*_1_)	Similarity factor (*f*_2_)
SR 3 core scale up	6.53	68.42
SR 3 subcoat	7.37	64.78
SR 3 film 1 h	11.72	56.62
SR 3 film 2 h	13.61	54.25
SR 3 film 3 h	13.32	54.23
SR 3 film 4 h	15.23	51.19
SR 10 core scale up	2.37	85.56
SR 10 subcoat	2.24	85.99
SR 10 film 1 h	6.23	72.19
SR 10 film 2 h	5.79	71.49
SR 10 film 3 h	6.74	69.30
SR 10 film 4 h	7.25	67.24

**Fig. 3:**
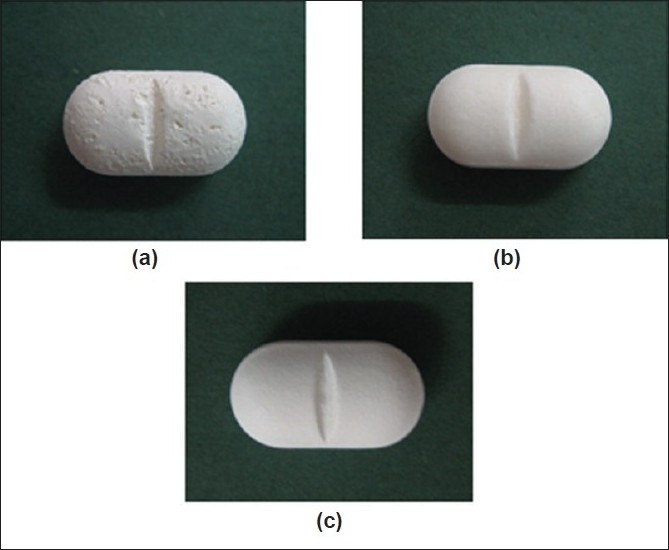
Photograph of scale-up SR 10 matrix tablet. Photograph of scale-up SR 10 matrix tablet after coated with: (a) HPMC-based film; (b) Eudragit^®^ L 30 D-55 subcoating film and (c) Eudragit^®^ L 30 D-55 subcoating film and over coated with HPMCbased film for 2 h at magnification of 10

There was the cratering defect which exhibited on tablet surface after coating with HPMC (fig. 3a). Therefore, the Eudragit^®^ L 30 D-55 subcoating of 0.5% by weight of total core tablet weight was performed by controlling the duration at 1 h of spraying. Triethyl citrate was added as a plasticizer at the concentration of 15% w/w of dry polymer weight. Subcoating with Eudragit^®^ L 30 D-55, an aqueous acrylic coating dispersion, has been employed for soft gelatin capsule[23]. After the subcoating process, the subsequent HPMC-based film coating could be performed without the appearance of crater. Coated matrix exhibited the smooth and homogeneous film after over coating with HPMC-based film.

**Fig. 4:**
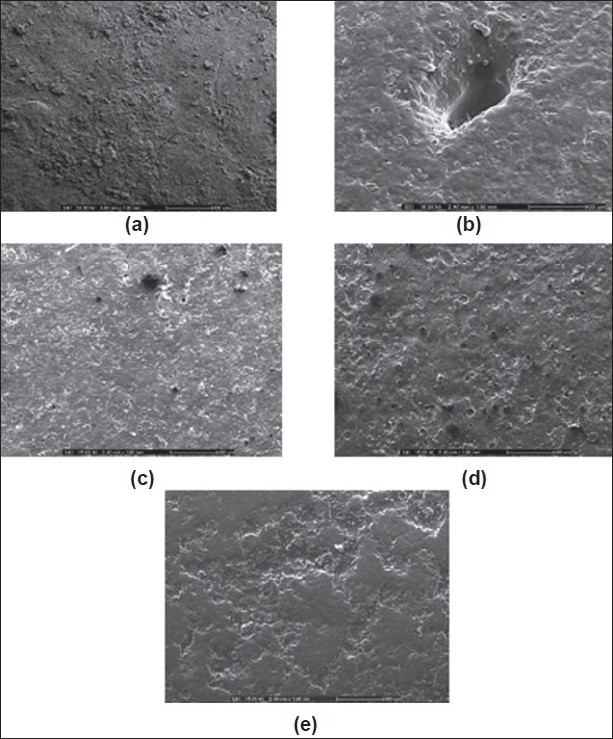
Scanning electron micrographs. Scanning electron micrographs of scale-up SR 10 matrix tablet surface morphology (50X) (a) the core tablet; (b) the cratering defect on matrix tablet after coated with HPMC film; (c) Eudragit^®^ L 30 D-55 subcoating film; (d) Eudragit^®^ L 30 D 55 film and over coated with HPMC-based film and (e) Depakine Chrono^®^ at magnification of 50

**Fig. 5:**
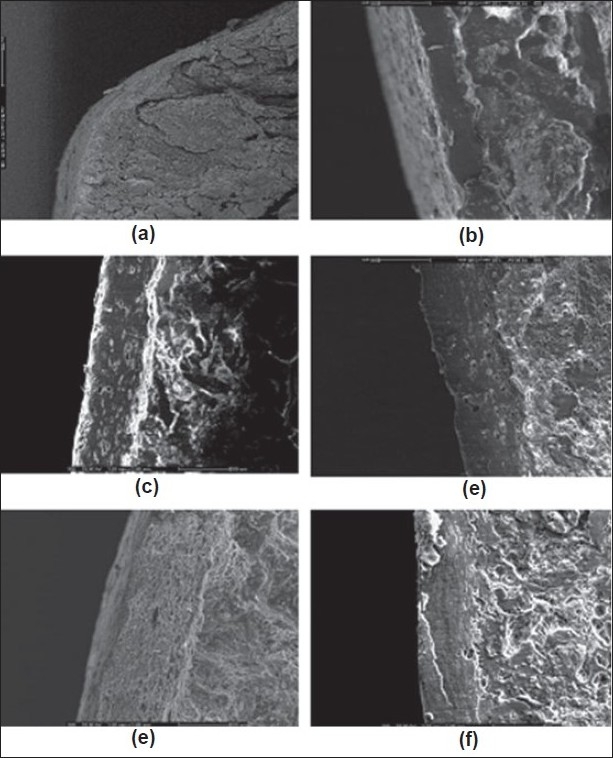
Scanning electron micrographs. Scanning electron micrographs of cross-section of scale-up SR 10 matrix tablets after different coating (100X) (a) Eudragit^®^ L 30 D-55 subcoating film; (b) Eudragit^®^ L 30 D-55 and over coated with HPMCbased film for 1 h; (c) 2 h; (d) 3 h; (e) 4 h and (f) Depakine Chrono^®^ at the magnification of 100

The cratering defect was evident when the directed spraying HPMC-based film was used (fig. 4b). This defect of film coating was volcanic-like craters on tablet surface. Because the coating solution penetrated the surface of the tablet, often at the crown where the surface was more crater, the localized disintegration of the core and disruption of the coating was exhibited[24]. This might be in line with an essentiality of VA which could be very soluble in organic coating solution in HPMC-based film. Therefore, a previous subcoating was applied to protect the penetration of HPMC-based coating solution into a core matrix tablet in this study. (Figs. 3b and 4c present the smooth subcoating with Eudragit^®^ L 30 D-55 and the lack of cratering defect. The surface of Depakine Chrono^®^ film (fig. 4e) was rather smooth and similar to that of the prepared matrix tablet as shown in fig. 4d. Scale-up core of SR10 matrix tablets were coated with Eudragit^®^ L 30 D-55 and over coated with HPMC-based film at different thicknesses by varying the spraying duration of HPMC solution (1, 2, 3 and 4 h). The thickness of film was increased as the duration of film coating was increased as presented in fig. 5a to e. The thin layer of Eudragit^®^ L 30 D-55 subcoating was evident (fig. 5a). The film thickness of Depakine Chrono^®^ (fig. 5f) was comparable to that of the matrix tablet coated with Eudragit^®^ L 30 D-55 and over coated with HPMC-based film for 2 h (fig. 5c).

**Fig. 6:**
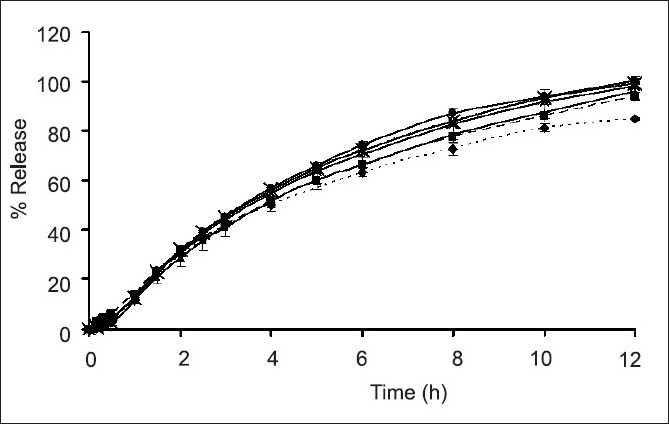
Comparative drug dissolution profile. Comparative drug dissolution profile of ‐‐■‐‐ scale up SR 3; ‐‐▲‐‐ scale up SR 3 after subcoat; scale up SR 3 after film coating for ‐‐×‐‐ 1 h; ‐‐◊‐‐ 2h; …×…. 3h; ‐‐♦‐‐ 4h and ….♦…. Depakine Chrono^®^ in phosphate buffer pH 6.2 (n=3)

**Fig. 7:**
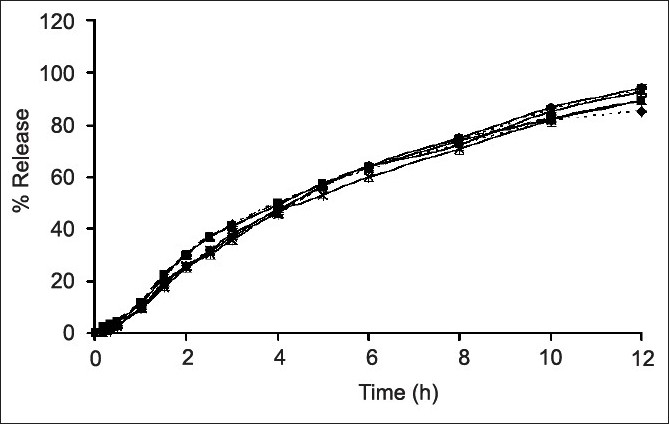
Comparative drug dissolution profile. Comparative drug dissolution profile of ‐‐■‐‐ scale up SR 10; ‐‐▲‐‐ scale up SR 10 after subcoat; scale up SR 10 after film coating for ‐‐×‐‐ 1 h; ‐‐◊‐‐ 2h; …×…. 3h; ‐‐♦‐‐ 4h and ….♦…. Depakine Chrono^®^ in phosphate buffer pH 6.2 (n=3)

The slight lag time in release profiles (figs. 6 and 7) was the time required for the dissolution medium to diffuse through the coating layer and for the dissolved drug molecules to diffuse outward across film coating[25]. The subcoating with Eudragit^®^ L 30 D-55 did not affect the drug release considering from drug release profiles of both the scale-up core tablets and the Eudragit^®^ L 30 D-55 subcoating tablets. The drug release rates of over coated HPMC-based film of SR3 were greater than that of SR10 due to the amount of HPMC K15M and microcrystalline cellulose in the core of each formula. SR10 contained rather high amount HPMC K15M, therefore the drug release was more retarded from the effect of added polymer. After the tablets contacted with the dissolution medium, water penetration between microcrystalline cellulose in the tablet and a local swelling occurred. Water was trapped in microcrystalline cellulose as a result of adsorption and capillary effects. Then, the crystalline framework burst and microcrystalline cellulose fragmented into smaller particles[26]. The amount of microcrystalline cellulose in SR3 formula was higher than that in SR10. Therefore, the drug release of SR3 was faster than SR10. The similarity factor (*f*_2_) was greater than 50 for all the film coated formula and the difference factor (*f*_1_) was less than 15 for most of film coated formula except SR3 film coated at 4 h which was 15.23 indicating the different drug release profiles from Depakine Chrono^®^Table 3.

There was a tendency of the slight increment of drug release rate as the over coated HPMC-based film thickness was increased. It was possible that a drug or a core component migrated in or onto an applied film during coating. It has been reported earlier that coating conditions could affect the water penetration to the substrate during the coating process and subsequently the migration of water soluble components of the tablet core to the film coating. If components of the core migrate into the film layer during the early stages of the coating process, it could lead to heterogeneous film formation[27]. Drug migration into polymeric film coat has been previously reported[28,29]. Since spraying period was short, it might not completely cover the surface of the core matrix tablets. However, the slight increase in drug release from the tablet after over coating with HPMC-based film might be due to the property of VA which could be very soluble in organic coating solution in HPMC-based film that supported the drug migration on polymer film. Although the core matrix tablets were coated with Eudragit^®^ L 30 D-55 which was generally used for enteric film coating, it was possible that the migrated drug could be released into 0.1 N hydrochloric acid.

From curve fitting, the drug release from tablets containing HPMC K 15 M was a diffusion control. The best fit model was the Higuchi's model since r^2^ and MSC from curve fitting were apparently higher than those of the first order and zero order curve fittings Table 4. The estimate parameters from curve fitting to power law equation were presented in Table 5. The high value of a model selection criteria (MSC) indicated the high degree of goodness-of-fit with power law equation.

**TABLE 4:** COMPARISON OF DEGREE OF GOODNESS-OF-FIT BETWEEN DIFFERENT RELEASE MODELS AND DISSOLUTION DATA

Tablet	First order		Higuchi's		Zero order	
	r^2^	MSC	r^2^	MSC	r^2^	MSC
Depakine chrono	0.9903	3.97	0.9986	5.88	0.9361	2.08
SR 1	0.9936	4.25	0.9997	7.34	0.9519	2.23
SR 2	0.9946	4.55	0.9993	6.55	0.9620	2.60
SR 3	0.9945	4.54	0.9994	6.77	0.9480	2.29
SR 4	0.9877	3.83	0.9973	5.33	0.9411	2.26
SR 5	0.9910	4.14	0.9960	4.94	0.9342	2.15
SR 6	n.d.	n.d.	n.d.	n.d.	n.d.	n.d.
SR 7	0.9920	3.83	0.9998	7.39	0.9788	2.85
SR 8	0.9944	4.69	0.9987	6.15	0.9608	2.74
SR 9	0.9550	2.6	0.9654	2.86	0.9911	4.23
SR 10	0.9970	5.25	0.9992	6.58	0.9587	2.61
SR 11	0.9989	6.03	0.9975	5.18	0.9920	4.03
SR 12	0.9700	2.84	0.9967	5.05	0.9581	2.51
SR 3 scale up core	0.9967	5.26	0.9980	5.75	0.962	2.83
SR 3 subcoating	0.9906	4.23	0.9958	5.02	0.9637	2.87
SR 3 film 1 h	0.9853	3.72	0.9956	4.94	0.9714	3.05
SR 3 film 2 h	0.9846	3.68	0.9964	5.14	0.9702	3.01
SR 3 film 3 h	0.9810	3.46	0.9964	5.12	0.9680	2.94
SR 3 film 4 h	0.9707	3.03	0.9933	4.51	0.9724	3.09
SR 10 scale up core	0.9934	4.58	0.9995	7.17	0.9498	2.55
SR 10 subcoating	0.9930	4.52	0.9994	7.03	0.9494	2.54
SR 10 film 1 h	0.9954	4.87	0.9978	5.63	0.9714	3.05
SR 10 film 2 h	0.9902	4.18	0.9917	4.35	0.9594	2.76
SR 10 film 3 h	0.9924	4.38	0.9961	5.04	0.9792	3.37
SR 10 film 4 h	0.9894	4.05	0.9956	4.93	0.9766	3.25

n.d. = not determined

**TABLE 5:** ESTIMATE PARAMETERS FROM CURVE FITTING WITH POWER LAW EQUATION

Tablet	k±SD ×10^-3^	tl±SD (min)	n±SD	MSC
Depakine chrono	41.6111±3.4633	51.42±1.72	0.4731±0.0144	6.29
SR 1	42.5115±2.1818	46.31±1.14	0.4849±0.0091	7.78
SR 2	31.2783±1.0793	44.72±0.93	0.5287±0.0058	8.50
SR 3	42.1782±0.8880	46.82±0.55	0.4751±0.0036	9.20
SR 4	46.0594±0.7651	47.56±0.49	0.4480±0.0027	9.36
SR 5	49.4263±2.5105	49.34±1.37	0.4418±0.0084	7.04
SR 6	n.d.	n.d.	n.d.	n.d.
SR 7	39.2524±1.8993	39.94±0.99	0.5246±0.0089	9.11
SR 8	50.2509±4.1183	44.92±2.20	0.4751±0.0145	6.32
SR 9	7.0828±4.5132	18.44±18.38	0.8026±0.1042	4.27
SR 10	28.1273±1.3173	46.39±1.38	0.5251±0.0076	7.67
SR 11	16.4512±1.7424	38.13±5.34	0.6042±0.0165	8.18
SR 12	69.7833±11.2683	47.47±3.17	0.4545±0.0310	5.17
SR 3 scale up core	30.2013±2.5710	44.58±2.52	0.5360±0.0144	6.35
SR 3 subcoating	26.7757±2.4023	46.70±2.47	0.5586±0.0152	6.26
SR 3 film 1 h	27.0354±2.4638	46.10±2.16	0.5690±0.0160	6.48
SR 3 film 2 h	28.7964±2.3300	46.38±1.91	0.5618±0.0142	6.68
SR 3 film 3 h	29.2102±2.3905	48.14±1.83	0.5599±0.0145	6.57
SR 3 film 4 h	25.5681±1.7129	48.62±1.45	0.5874±0.0118	7.06
SR 10 scale up core	37.0145±1.8662	49.76±1.29	0.4955±0.0087	6.99
SR 10 subcoating	36.8874±1.9845	49.91±1.37	0.4953±0.0093	6.85
SR 10 film 1 h	28.7273±4.5260	60.53±5.78	0.5319±0.0260	5.69
SR 10 film 2 h	23.9260±4.2564	48.22±4.65	0.5663±0.0302	4.87
SR 10 film 3 h	23.3955±4.0753	53.38±6.66	0.5721±0.0284	5.82
SR 10 film 4 h	24.4961±4.8292	56.61±7.33	0.5681±0.0322	5.50

n.d. = not determined

The values of exponent (n) for most of the formulations were shown in Table 5. For a matrix tablet, a cylindrical geometry was considered; n takes values in the range of 0.45-0.89 for anomalous transport[22]. The high water uptake, leading to higher swelling of the tablet, supported the anomalous release mechanism of VA. The n value of the optimized formulation (scale-up SR 10 core) was found to be 0.4955 while that of the marketed formulation was 0.4731, indicating the Fickian diffusion or nearly tended to Fickian diffusion (n=0.45). While the matrix tablet came into contact with a dissolution medium, the macromolecular chains of HPMC swelled at the tablet surface and formed a gel layer around a dry-like core. Drug diffusion occurred at the core-gel interface then through this gel[26]. The erosion of the swollen layer and the dissolution of the matrix itself were also observed. The drug release data were explored for the release mechanisms that followed. For the controlled or sustained release formulations, the diffusion, swelling and erosion were the three most important rate-controlling mechanisms. The drug release from the polymeric system was mostly occurred by diffusion and was best described by the Fickian diffusion. In conclusion, the VA and VAS sustained-release matrix tablets were prepared using HPMC as a matrix former which could prolong the drug release for 12 h. Aerosil^®^ 200 effectively adsorbed oily VA and slightly influenced the drug release of the matrices. The drug release from optimized formulation followed the Higuchi's kinetics while the mechanism of drug release was the Fickian diffusion, controlled by diffusion through a swollen matrix. Eudragit^®^ L 30 D-55 was used as subcoating material for scale-up core matrix tablets before over coating with HPMC-based film. The similarity factor (*f*_2_) and difference factor (*f*_1_) values of drug release profile of scale-up SR10 after film coating were greater than 50 and were less than 15, respectively, supporting the similar release to that of Depakine Chrono^®^.
